# Isolation of *Cutibacterium acnes* AP1, a rumen bacterium that forms *trans-*10,*cis-*12-conjugated linoleic acid

**DOI:** 10.3168/jdsc.2024-0598

**Published:** 2024-08-16

**Authors:** Timothy J. Hackmann, Marcelo Saldivia, Lynn Wolfe, Hannah De Groot, Jingyi Yang, Payam Vahmani

**Affiliations:** Department of Animal Science, University of California, Davis, Davis, CA 95168

## Abstract

Some microbes in the rumen form *trans-*10,*cis-*12 (*t*10,*c*12)-conjugated linoleic acid (CLA), a fatty acid that depresses synthesis of milk fat in dairy cattle and other lactating animals. Despite their importance to milk fat depression, the microbes responsible have been difficult to identify, and no laboratory strain is currently available for study. Here we describe the isolation of *Cutibacterium acnes* AP1, a bacterium that forms *t*10,*c*12-CLA at fast rates. It was isolated from rumen contents of a heifer by using medium containing lactate and metronidazole. It formed *t*10,*c*12-CLA from linoleic acid, a result confirmed by a combination of 3 methods (spectrophotometry, thin-layer chromatography, and GC). The initial rate of formation was 67% faster than for *Cutibacterium acnes* DSM 1897, the type strain from human skin. The availability of this isolate and its ability to rapidly form *t*10,*c*12-CLA make it a good subject for further study. Such study could help control formation of *t*10,*c*12-CLA in the rumen and alleviate milk fat depression in milk cows.

## Highlights

• Some rumen microbes form *t*10,*c*12-CLA, which depresses dairy cow milk fat.
• Despite the importance of these microbes, no laboratory strain is available.
• Here, we isolate Cutibacterium acnes AP1, a rumen bacterium that forms *t*10,*c*12-CLA.
• *C. acnes* forms *t*10,*c*12-CLA, doing so 67% faster than a bacterium from human skin.
• Studying *C. acnes* AP1 will be key to alleviating milk fat depression in cows.

## Graphical Abstract

**Figure:**
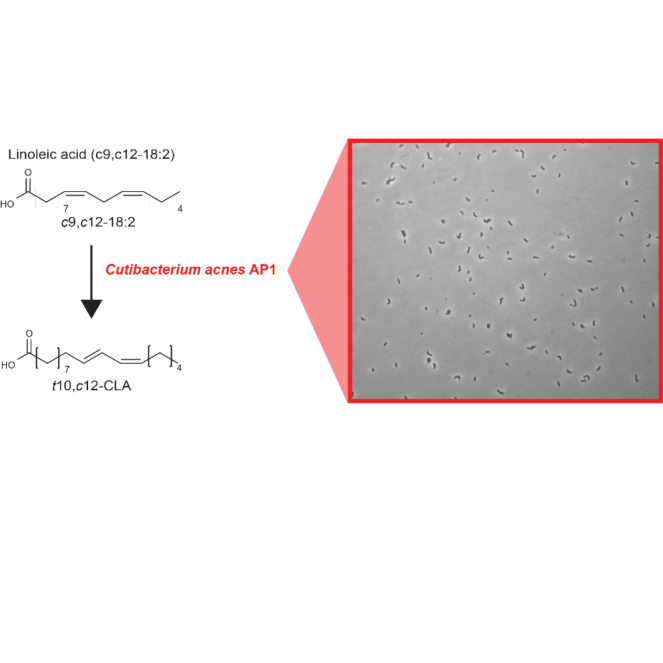
**Summary:** Some microbes in the rumen form trans-10,*cis*-12 (*t*10,*c*12)-conjugated linoleic acid (CLA), and this compound depresses milk fat synthesis by the cow. Because unintentional cases of milk fat depression cause large losses of milk fat, we need laboratory strains of these microbes for further study, but no strain from the rumen is currently available. Here, we isolate 1 bacterium, *Cutibacterium acnes* AP1, that can form *t*10,*c*12-CLA. Cultures of this organism form *t*10,*c*12-CLA from linoleic acid, and the initial rate of formation is 67% faster than *C. acnes* DSM 1897, a related bacterium from human skin. Studying *C. acnes* AP1 and other organisms that form *t*10,*c*12- CLA will be key to alleviating milk fat depression in cows. There are circumstances in which milk fat depression could be beneficial, and our results could also be important in inducing milk fat depression.

Milk fat depression is a costly nutritional disorder of dairy cattle and other lactating animals. It occurs when feeding diets high in fermentable carbohydrate or unsaturated fat, and it depresses yield of milk fat by up to 50% (Bauman and Griinari, 2003). Though nutritionists are aware of the disorder, field studies confirm that it is still present on many dairy farms (McCarthy et al., 2018). With fat accounting over 50% of the price of milk (USDA-ERS, 2024), losses from unintentional cases of milk fat depression are large.

To alleviate the disorder, it is important to consider the role of microbes in the rumen. Certain rumen microbes form *trans-*10,*cis-*12 (*t*10,*c*12)-CLA and other antilipogenic fatty acids during the process of biohydrogenation (Dewanckele et al., 2020b). These antilipogenic fatty acids can be absorbed from the rumen and circulate to the mammary gland, where they depress milk fat synthesis (Bauman and Griinari, 2003). As a result, it is important to study these microbes and how the diet can trigger them to form antilipogenic fatty acids.

Despite the importance of microbes forming *t*10,*c*12-CLA, there are currently no strains available to study in the laboratory. One strain was thought to be *Megasphaera elsdenii* T81 (Kim et al., 2002). Along with another strain (YJ-4), it was found to form *t*10,*c*12-CLA at fast rates. However, later work found this strain does not form *t*10,*c*12-CLA (Maia et al., 2007; Dewanckele et al., 2020a), and cultures were found to be contaminated with a nonrumen organism (Maia et al., 2007). *Cutibacterium acnes* G449 is a second strain found to form *t*10,*c*12-CLA (Wallace et al., 2007). This strain has since been lost, and investigators have been using a strain from human skin (*C. acnes* DSM 1897) as a substitute (Dewanckele et al., 2020a). While the strain from skin is the type strain and well studied, it may have properties that differ from rumen strains. Despite searching by several labs, no additional strains from the rumen have been found to form *t*10,*c*12-CLA.

In this work, we report isolation of a new strain of *C. acnes* from the rumen, and it forms *t*10,*c*12-CLA at fast rates. The existence of this organism was briefly mentioned in previous work (Hackmann and Vahmani, 2023), and it was isolated alongside other organisms, which have been described separately (Hackmann and Zhang, 2023). Here we describe isolation procedures specific to this organism and report some of the organism's characteristics.

To isolate the strain, we collected rumen contents from a Holstein heifer. The heifer was fed a normal diet consisting of 50.6% wheat hay, 24.6% alfalfa hay, 21.1% almond hulls, and 3.7% UC Davis Dry Cow Pellet (Western Milling LLC; DM basis). As determined by wet chemistry methods (Cumberland Valley Analytical Services), chemical composition was 91.8% OM, 43.8% NDF (analyzed with heat stable amylase and expressed exclusive of residual ash; aNDFom), 31.2% ADF, 12.7% CP, and 6.4% starch (DM basis). Contents from the rumen were collected through a fistula approximately 5 h after feeding, and serial dilutions were made with propionibacterium diluent (Hackmann and Zhang, 2023). All subsequent procedures were done anaerobically under O_2_-free CO_2_. Aliquots (0.1 mL) of each dilution were dispensed into anaerobic bottle plates containing 9 mL of LH medium (Mackie and Heath, 1979; Hackmann and Zhang, 2023). This medium was previously shown to support growth of propionibacteria, which includes *Cutibacterium* species, and other lactate-utilizing bacteria (Mackie and Heath, 1979). After discovering the medium supported growth of many nontarget bacteria, we added metronidazole (256 µg/mL media), an antibiotic to which propionibacteria are resistant (Sutter and Finegold, 1976). After incubation at 37°C for 7 d, isolated colonies were picked and transferred to Balch tubes containing 9 mL of LH medium. The 16S rRNA gene was amplified by PCR using 27f and 1492r primers (Lane, 1991) and subjected to Sanger sequencing (UCDNA Sequencing Facility). Sequences of relatives (propionibacteria) were downloaded from LPSN (Meier-Kolthoff et al., 2022) and aligned with Clustal Omega (Sievers et al., 2011). Following Hackmann (2022), a phylogenetic tree was built with W-IQ-TREE (Trifinopoulos et al., 2016) and visualized with ggtree (Yu et al., 2017).

We isolated *C*. *acnes* AP1 from a bottle inoculated with a 10^3^ dilution of rumen contents. Its cells were irregular rods and resembled *C*. *acnes* DSM 1897, which we used as an aid for initial identification. Sequencing of the 16S rRNA gene confirmed AP1 was a strain of *C*. *acnes*. A phylogenetic tree showed its closest relatives were *C*. *acnes* DSM 1897 and JCM 6473, which are different subspecies of this species (Figure 1). In addition to *C*. *acnes* AP1, we isolated several strains of *Propionibacterium australiense*, which have been previously isolated from lesions of rumen tissue (Bernard et al., 2002) but not rumen contents. We also isolated many non-propionibacteria and described 2 of those strains previously (Hackmann and Zhang, 2023). In total, 152 colonies from 40 bottles were picked before identifying *C*. *acnes* AP1.

**Figure 1:**
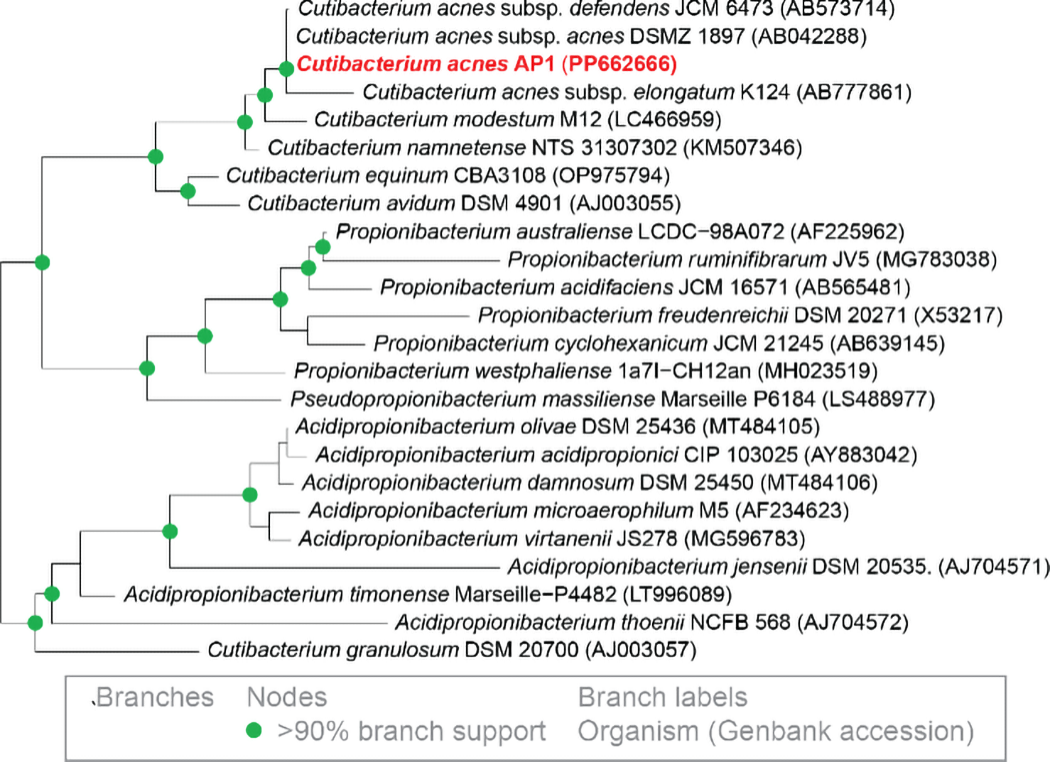
Strain AP1 belongs to *Cutibacterium acnes*. Phylogeny of the 16S rRNA gene places AP1 next to 2 subspecies of *C. acnes*. The sequence for *C*. *acnes* AP1 was determined in this study. A sequence for DSM 1987 was also determined in this study (GenBank PP662667), and it has 100% identity with the previously available sequence (GenBank AB042288).

To determine if *C*. *acnes* AP1 formed *t*10,*c*12-CLA, we grew it in the presence of linoleic acid and prepared culture samples for analysis. We prepared Balch tubes containing 9 mL of PYG medium (DSMZ medium 104) (Zhang et al., 2021) and 1 mL of linoleic acid solution. The latter solution contained 5 mg linoleic acid/mL in 1% wt/vol Tween 80, giving a final concentration of 0.5 g linoleic acid/L medium. This final concentration was the same as in Barrett et al. (2007). Tubes were inoculated with a 0.1-mL volume of a previous culture and incubated at 39°C. Growth was monitored by measuring optical density at 600 nm (**OD_600_**) on a spectrophotometer (Genesys 20, Thermo Fisher Scientific). At time points indicated in figures, we took aliquots of culture and removed cells by centrifugation (21,100 × *g* for 10 min at 4°C; 75003424 rotor and Legend Micro 21R centrifuge; Thermo Fisher Scientific).

We analyzed culture samples for presence of *t*10,*c*12-CLA with 3 different methods. The first method, spectrophotometry, was performed following Barrett et al. (2007). We extracted lipids by combining 1 mL of culture sample with 2 mL of isopropanol, vortexing, adding 1.5 mL of hexanes, and vortexing again. After waiting 5 min, we collected the upper (hexanes) layer. We placed 0.75 mL of this extract in quartz cuvettes (Hellma 114F-10–40) and measured the absorbance spectrum on a Molecular Devices M3 spectrophotometer. We included a standard of purified *t*10,*c*12-CLA (Nu-Chek Prep UC-61-A; 90% purity according to the manufacturer). The standard was prepared as a 0.3 g/L solution in PYG media and extracted in isopropanol and hexanes identically to the samples. The spectrum of samples and the standard were adjusted by subtracting the spectrum of PYG media (extracted in isopropanol and hexanes).

For the second method, silver-ion thin-layer chromatography (**Ag-TLC**), we extracted lipids as before. We then concentrated 2.5 mL of extract by evaporating hexanes at 40°C under N_2_ and resuspending it in 50 µL of new solvent. We applied 10 µL of concentrated extract to a TLC plate (20 cm height, Silica Gel 60 F254, 0.2 mm layer thickness; MilliporeSigma 1055540001). The plate was previously washed in solvent [chloroform-acetic acid (99:1 vol/vol)], soaked in AgNO_3_ in methanol (1% wt/vol), and activated at 105°C for 30 min. The plate was developed in the same solvent [chloroform-acetic acid (99:1 vol/vol)] to approximately 90% of its total height, dried, and developed again (for a total of 2 rounds of development). To prevent darkening of the plate, all steps were done under low or no light. Spots were visualized by dipping the plate in H_2_SO_4_-ethanol (1:9 vol/vol) and heating at 115°C for 5 min. Images were captured with a gel imager (Analytik Jena UVP ChemStudio Plus). For each plate, we included a standard of 18 µg of *t*10,*c*12-CLA (Nu-Chek Prep UC-61-A) and 18 µg of linoleic acid. The standard was prepared as a 9 g/L solution of each fatty acids in hexane, 2 µL of each was applied to the plate, and hexanes were evaporated.

For the third method, GC, we prepared 9-mL culture samples. Samples were freeze-dried, then fatty acids were converted to methyl esters by base-acid methylation with sodium methoxide followed by methanolic HCl (Alves et al., 2013). In early experiments, we used acid methylation, but this led to extensive isomerization of known CLA isomers. We also attempted to use base methylation, but this resulted in poor detection of methyl esters (likely due to poor conversion of free fatty acids to their methyl ester form). An internal standard (*c*10–17:1 methyl ester; Nu-Chek Prep U-42-M) was added before methylation. The gas chromatograph was a Trace 1310 equipped with AI 1310 autosampler, split/splitless injector, and flame ionization detector (**FID**; Thermo Scientific). The column was a CP-Sil 88 (100 m length, 0.25 mm inside diameter, coated with a 0.2-μm film thickness; Agilent). The carrier gas was H_2_ (1 mL/min). The injection was performed in split mode (20:1 split). The front inlet had a temperature of 250°C. The oven temperature was initially 45°C, maintained for 4 min, raised to 175°C at 13°C/min, held at 175°C for 27 min, raised to 215°C at 4°C/min, and finally held at 215°C for 35 min (Kramer et al., 2008). The FID had a temperature of 250°C and flow rates for air, hydrogen, and nitrogen of 300, 30, and 29 mL/min, respectively. The injected sample volume was 1 μL, and the total run time for each analysis was 86 min. The standard was 0.5 mg of *t*10,*c*12-CLA (Larodan 10–1826; >98% purity according to the manufacturer). This standard was prepared as a 10 g/L solution in ethanol; 50 µL was dispensed into a tube, ethanol was evaporated, and fatty acids were freeze-dried and methylated identically to samples. The amount gave a final concentration equivalent to 0.0556 g/L medium. In addition to this standard, we used commercial fatty acid mixtures (Nu-Chek Prep GLC-463 and GLC-603) and a reference fat to identify more fatty acids. The reference fat was backfat from a steer fed a 75% alfalfa-grass hay and 25% flaxseed-based concentrate diet (Vahmani et al., 2017). Fatty acids in this reference were themselves identified by comparing their retention times and elution order to samples previously characterized by the authors (Vahmani et al., 2016) and reported in the literature (Cruz-Hernandez et al., 2004; Kramer et al., 2008; Fuentes et al., 2009).

The combination of these 3 methods of analysis suggested that *C*. *acnes* AP1 formed *t*10,*c*12-CLA. When we analyzed samples with spectrophotometry, it revealed a product with absorbance spectrum similar to *t*10,*c*12-CLA (Figure 2). This compound absorbs maximally at 233 nm due to a conjugated double bond, as observed with other isomers of CLA (Kepler and Tove, 1969). When we analyzed samples with Ag-TLC, we found a spot with the same retention factor (*R_f_*) as *t*10,*c*12-CLA (Figure 2). Separating *t*10,*c*12-CLA from linoleic acid was challenging at first, but good separation was afforded by 2 rounds of development in solvent. We did not establish if this method separates *t*10,*c*12-CLA from other isomers of CLA. Finally, GC showed a product with the same retention time as *t*10,*c*12-CLA (Figure 2). A second CLA isomer (*t*10,*t*12-CLA) was also detected with GC (Figure 2), but only in small amounts, and we cannot rule it out being formed during base-acid methylation. A third isomer (*c*9,*t*11-CLA) was detected, but its concentration did not increase from 0 to 48 h, indicating no net formation. The same products were also detected with *C. acnes* DSM 1897 (not shown). In previous studies, *C. acnes* DSM 1897 was identified to form *t*10,*c*12-CLA with TLC-MS (Verhulst et al., 1987) and GC-MS (Deng et al., 2007; Dewanckele et al., 2020a). These methods were not available to us, but our finding that DSM 1897 and AP1 form similar products supports that the latter forms *t*10,*c*12-CLA. Together, these results support that *C*. *acnes* AP1 forms *t*10,*c*12-CLA from linoleic acid.

**Figure 2:**
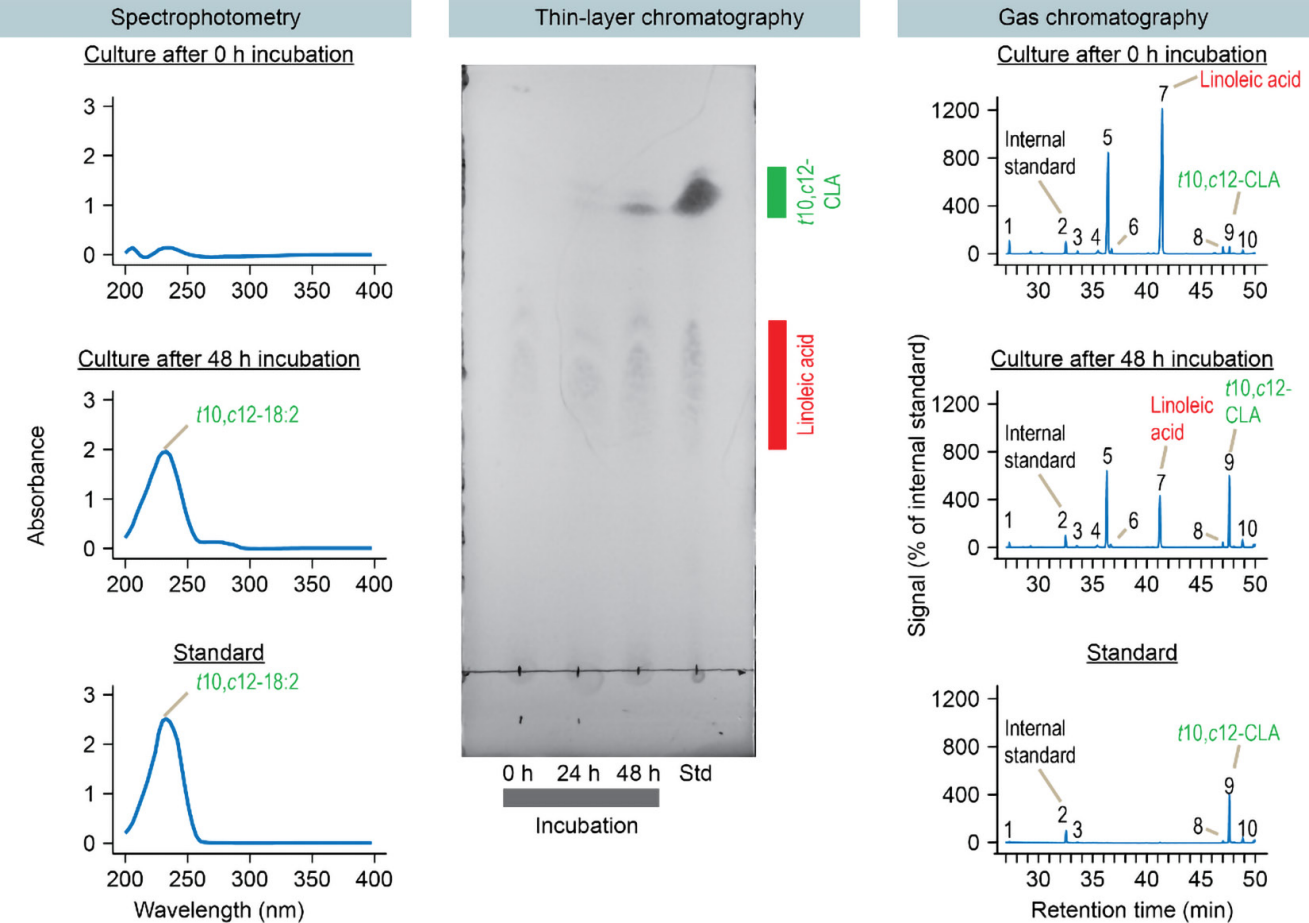
Strain AP1 converts linoleic acid to *t*10,*c*12-CLA according to spectrophotometry, silver-ion thin-layer chromatography (Ag-TLC), and GC. Peaks for GC are (1) C16:0, (2) *c*10–17:1, (3) C18:0, (4) *t*10–18:1, (5) *c*9–18:1, (6) *c*11–18:1, (7) linoleic acid (C18:2n-6), (8) *c*9,*t*11-CLA, (9) *t*10,*c*12-CLA, and (10) *t*10,*t*12-CLA. Peaks shown are those that account for >1% of total fatty acids (according to peak area) in at least one sample. Results are representative of 3 biological replicates (samples prepared from independent cultures). Std = standard.

We next wanted to measure how quickly *C*. *acnes* AP1 forms *t*10,*c*12-CLA and how it compares with *C. acnes* DSM 1897, the type strain from human skin. We obtained *C. acnes* DSM 1897 from the DSMZ. We grew both strains in tubes containing PYG with linoleic acid (0.5 g/L medium) and collected samples at time points indicated in figures. We analyzed extracts by spectrophotometry as before, except we used plastic cuvettes (Brandtech 759165). Concentrations of *t*10,*c*12-CLA were determined using pure *t*10,*c*12-CLA (Nu-Chek Prep UC-61-A) in PYG medium as an external standard. The standard contained between 0 to 0.5 g/L *t*10,*c*12-CLA. Samples and standards were diluted with hexanes to ensure absorbance stayed within the linear range of the instrument. As a control, we grew strains in tubes in PYG without linoleic acid. To maximize growth and production of *t*10,*c*12-CLA, each strain was transferred daily (every 24 h). Stable growth and *t*10,*c*12-CLA formation was achieved after 3 transfers. We analyzed data for growth and formation of *t*10,*c*12-CLA using a linear model. We used the nlme package of R, the gls function, and model Value ∼ Strain*Linoleic_acid*Time. This model includes fixed effects of strain, linoleic acid, and incubation time, and their interaction. To reflect the repeated measures design, culture tube was the grouping factor. When analyzing formation of *t*10,*c*12-CLA, we chose an unstructured covariance matrix. When analyzing growth, we chose an autoregressive structure with unequal variance instead. In each case, the covariance structure chosen was the one which gave the lowest value of Akaike information criterion (AIC). Values of SEM were estimated using the emmeans package of R. We tested whether means differed from each other using a 2-tailed *t*-test, and we corrected *P*-values for multiple comparisons with the Tukey procedure.

We found that *C*. *acnes* AP1 grew and formed *t*10,*c*12-CLA at fast rates (Figure 3A). Over 48 h, it grew to 63% higher density (OD_600_) than *C. acnes* DSM 1897. It also formed 67% more *t*10,*c*12-CLA over the same period. By 72 h, *C*. *acnes* AP1 had converted 99.6% (3.7% SEM) of the original dose of linoleic acid to *t*10,*c*12-CLA; the value for *C. acnes* DSM 1897 was similar [94.6% (3.7% SEM)]. When linoleic acid was absent, formation of *t*10,*c*12-CLA was minimal. For *C. acnes* DSM 1897, presence of linoleic acid resulted in lower growth at 48 h, but *C*. *acnes* AP1 was not affected by it (Figure 3A). These experiments underscore the ability of *C*. *acnes* AP1 to grow in presence of linoleic acid and form *t*10,*c*12-CLA.

**Figure 3:**
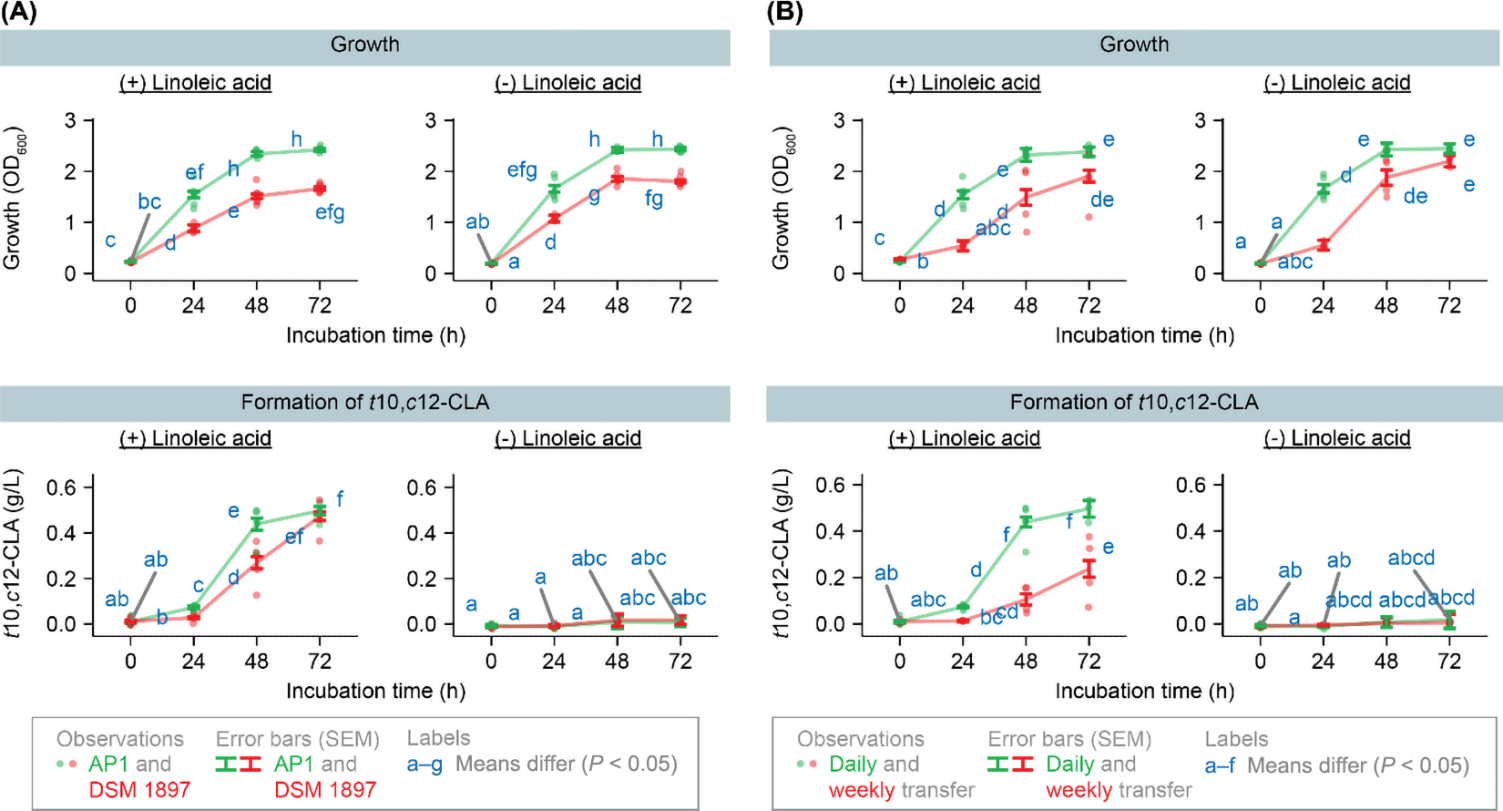
Strain AP1 forms *t*10,*c*12-CLA at a fast rate, which is linked to its fast growth. (A) Growth and formation of *t*10,*c*12-CLA are initially faster than strain DSM 1897, the type strain from human skin. For growth, results are from 5 biological replicates (independent cultures). For formation of *t*10,*c*12-CLA, results are from 3 biological replicates at 72 h and 5 biological replicates at other incubation times. (B) Growth and formation of *t*10,*c*12-CLA are slower when the culture is transferred weekly instead of daily. For weekly transfer, results are from 4 biological replicates. For daily transfer, data are from panel A, and number of replications is as stated above.

It appeared that *C*. *acnes* AP1 formed *t*10,*c*12-CLA at a fast rate because of its fast growth. To explore this idea, we intentionally slowed growth of this strain by transferring it weekly (every 168 h). We combined these data with those from Figure 3A (where the strain was transferred daily) and analyzed the data using the model Value ∼ Transfer_interval*Linoleic_acid*Time. This model is similar to that above, except it included the fixed effect of transfer interval.

These set of experiments confirmed that formation of *t*10,*c*12-CLA was linked to growth rate. Transferring the culture every week successfully slowed the growth rate, and this resulted in slower biohydrogenation (Figure 3B). These results underscore that growth conditions influence biohydrogenation, and they suggest faster growth may partly explain why *C*. *acnes* AP1 formed *t*10,*c*12-CLA faster than *C. acnes* DSM 1897.

The isolation of *C*. *acnes* AP1 should help in the study of how rumen microbes form *t*10,*c*12-CLA. At present, formation of *t*10,*c*12-CLA is studied with *C. acnes* DSM 1897—it is a model organism used by rumen microbiologists (Wallace et al., 2007; Dewanckele et al., 2020a) and others (Verhulst et al., 1987; Deng et al., 2007). However, *C. acnes* DSM 1897 is from human skin, and it forms *t*10,*c*12-CLA more slowly than the strain from the rumen. One potential application, which we have previously proposed, is using *C*. *acnes* AP1 to screen enzyme inhibitors (Hackmann and Vahmani, 2023). These inhibitors could be used to slow formation of *t*10,*c*12-CLA by this strain and others in the rumen and alleviate milk fat depression.

While the current work is an important step, it is important to obtain more strains forming more antilipogenic fatty acids. We isolated the current strain with difficulty, reflecting that *C. acnes* is not highly abundant in the normal rumen (Shingfield et al., 2012). While our strain was isolated from the rumen of a heifer, it would be important to isolate strains from cows with milk fat depression. Likewise, there are antilipogenic acids in addition to *t*10,*c*12-CLA, though evidence for activity of *t*10,*c*12-CLA is best (Dewanckele et al., 2020b). While we have treated formation of *t*10,*c*12-CLA and milk fat depression as undesirable, there are circumstances where they could be beneficial. These circumstances could include during shortage of feed, breeding, and other periods where negative energy balance needs to be mitigated (Harvatine et al., 2009). The discovery of more strains forming *t*10,*c*12-CLA would be just as important to inducing milk fat depression as alleviating it. The methods we outline here should guide efforts to identify more of these strains.
